# Supplementary material for: The GATA-Type Transcriptional Factor Are1 Modulates the Expression of Extracellular Proteases and Cellulases in Trichoderma reesei
Source: Int J Mol Sci. 2019 Aug 22;20(17):4100. doi: 10.3390/ijms20174100 (PMC6747117; doi:10.3390/ijms20174100)
Supplement: Supplementary file 1 [file ijms-20-04100-s001.pdf]

Supplementary materials

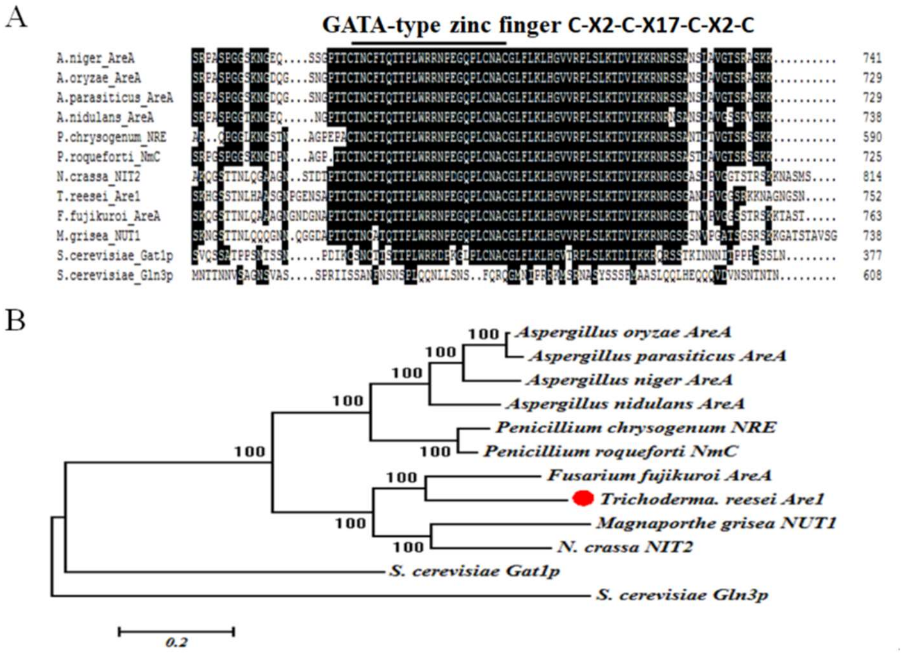

Figure S1. Sequence comparison of the *T. reesei* Are1 protein with related sequences. (A) Alignment of the amino acid sequence of the *T. reesei* Are1 with related fungal sequences. Identical amino acids are indicated with black backgrounds. (B) Phylogenetic tree of Are1 homologs in various species. Sequence accession numbers are as follows: *A. nidulans* AreA (CAA36731.1); *A. niger* AreA (CAA68196.1); *A. oryzae* AreA (AAK08066.1); *A. parasiticus* AreA (AAD37409.1); *F. fujikuroi* AreA (CAA71897.1); *M. grisea* NUT1 (AAB03415.1); *N. crassa* NIT2 (P19212.2); *P. chrysogenum* NRE (AAA83400.1); *P. roqueforti* Nmc (CAA04815.1); *S. cerevisiae* Gat1p (KZV11580.1) and Gln3p (KZV11792.1).

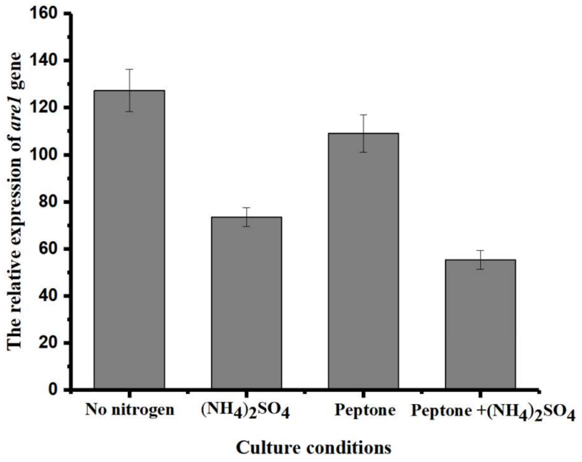

Figure S2. The transcript levels of *are1* in *T. reesei* QM9414 under different culture conditions.

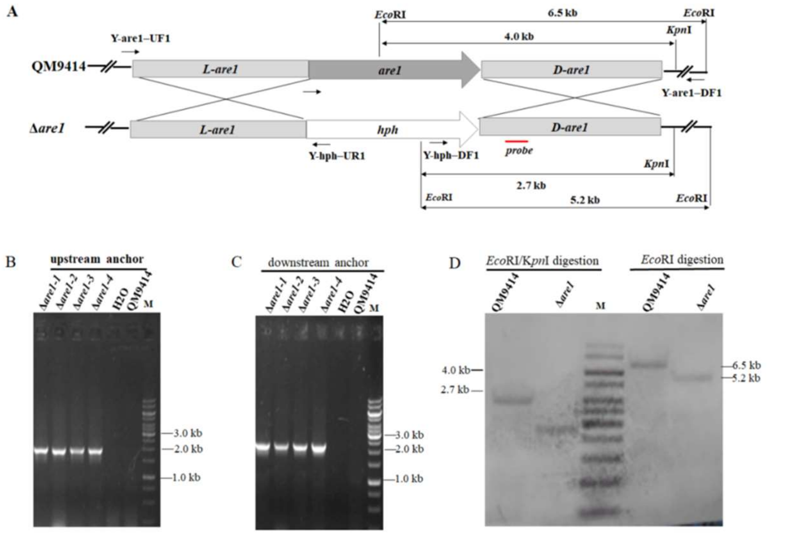

Figure S3. Graphical representation of the *are1* genomic locus in the *T. reesei* strains QM9414 and Δ*are1*. Primer pairs and relative positions of the E*coR*I and K*pn*I restriction sites are given. Probe used for Southern analysis is shown as red box. (B) and (C) PCR analysis of the transformants showed that the upstream and downstream of the *are1* gene had been anchored by gene deletion cassette using *hph* as a selection marker. (D) Southern blot of the chromosomes digested with E*coR*I and K*pn*I using a fragment of the *are1* gene as the probe.

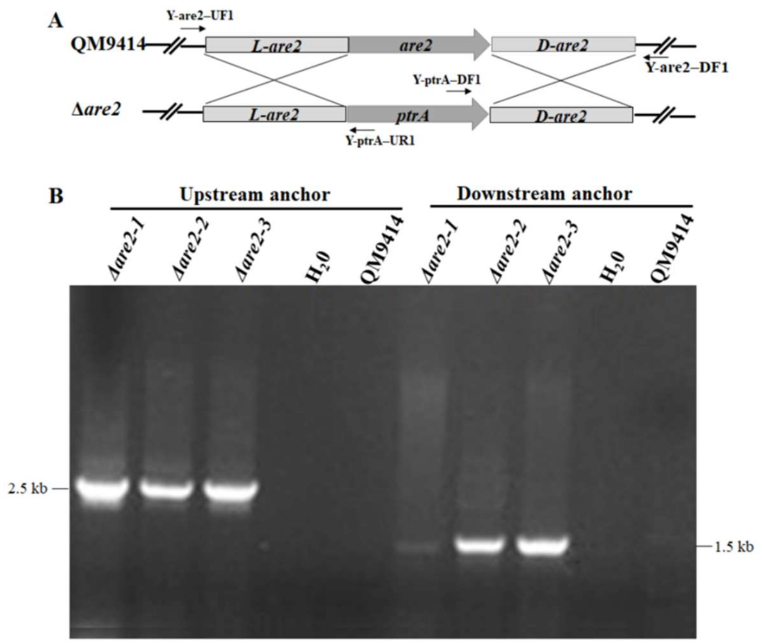

Figure S4. Deletion of *are2* in *T. reesei*. (A) Schematic representation of the genomic organization of the *are1* locus in QM9414 and Δ*are2*. Primer pairs for PCR analysis are indicated to produce the expected fragment size. (B) PCR analysis of transformants with primer pairs Y-are2-UF1/Y-hph-UR1 (for upstream anchor) and Y-hph-DF1/Y-are2-DR1 (for downstream anchor), which should produce the PCR products of 2.5kb and 1.5kb, respectively.

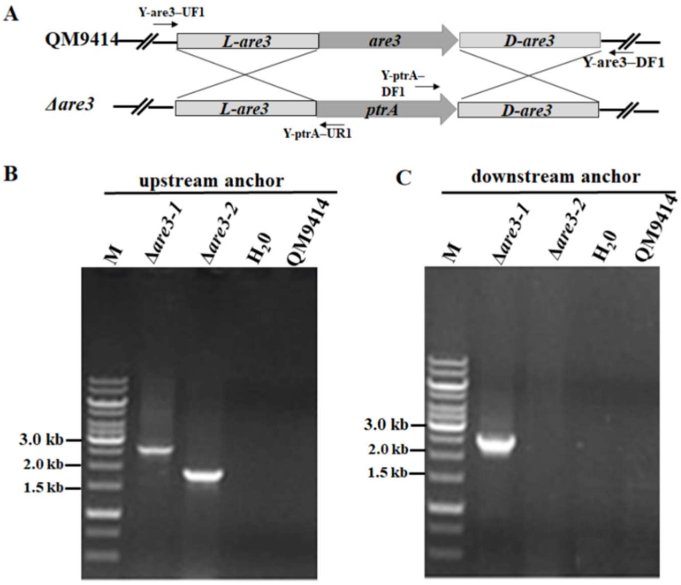

Figure S5. Deletion of *are3* in *T. reesei*. (A) Schematic representation of the genomic organization of the *are3* locus in QM9414 and Δ*are3*. Primer pairs for PCR analysis are indicated to produce the expected fragment size. (B) and (C) PCR analysis of transformants with primer pairs Y-are3-UF1/Y-hph-UR1 (for upstream anchor) and Y-hph-DF1/Y-are3-DR1 (for downstream anchor), which should produce the PCR products of 2.6kb and 2.2kb, respectively.

.

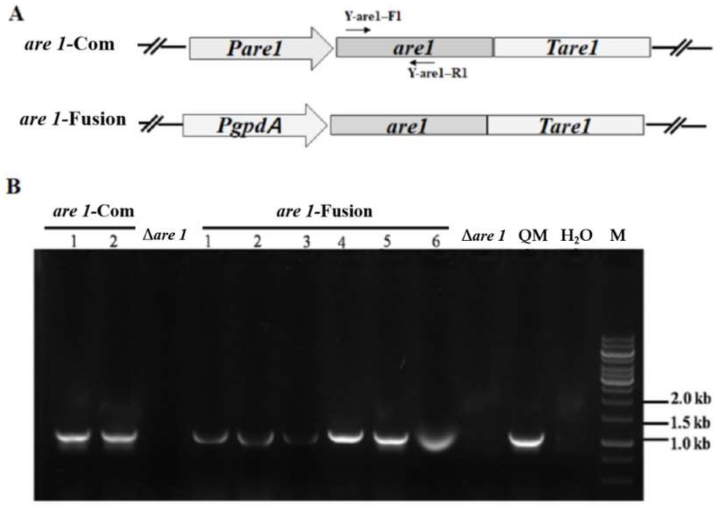

Figure S6. Complementation of *are1*. (A) Schematic representation of the genomic organization of the *are1* locus in the *are1*-Com and *are1*-Fusion strains. (B) PCR analysis of the *are1* gene using the primer pair Y-are1-F1/Y-are1-R1 in the *are1*-Com and *are1*-Fusion strains, which should produce the PCR product of 1.1kb.
